# Supplementary material for: Quantification of Structural Integrity and Stability Using Nanograms of Protein by Flow-Induced Dispersion Analysis
Source: Molecules. 2022 Apr 13;27(8):2506. doi: 10.3390/molecules27082506 (PMC9027858; doi:10.3390/molecules27082506)
Supplement: Supplementary file 1 [file molecules-27-02506-s001.zip › molecules-1630801-supplementary.pdf]

Supplementary information

Quantification of Structural Integrity and Stability using Nanograms of Protein by Flow Induced Dispersion Analysis

Morten E. Pedersen[1,2], Jesper Østergaard[2], and Henrik Jensen[1]

[1] Fida Biosystems ApS, Generatorvej 6, 2860 Soeborg, Denmark.

[2] Department of Pharmacy, University of Copenhagen, Universitetsparken 2, 2100 Copenhagen O, Denmark.

Representative raw data for protein unfolding using capillary mixing mode

Figure S1 demonstrates raw data (taylorgrams) for adalimumab denaturation at pH 10 obtained with capillary mixing. Peak broadening was ascribed to unfolding of adalimumab at increasing concentrations of GuHCl. Data treatment of the fluorescence dips in the peak center is described in the following section.

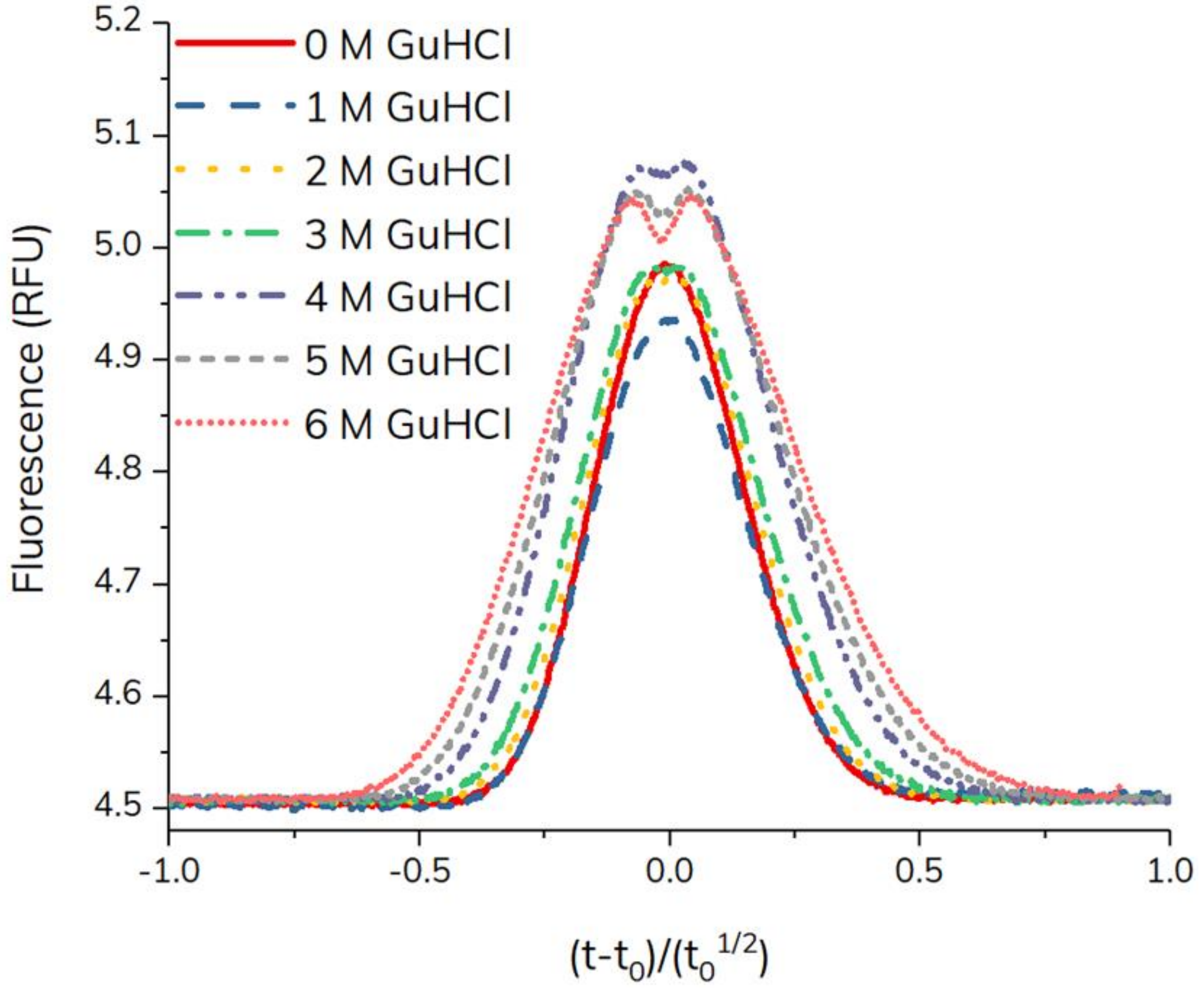

Figure S1: Taylorgrams (i.e., raw data) for adalimumab (1 mg/mL) at pH 10.0 in presence of increasing concentrations of GuHCl (0-6 M). Normalized for changes in viscosity as per [26].

Data analysis of mismatch taylorgrams

The capillary mixing mode led to irregular taylorgrams, see Figure S2. Others have ascribed this type of taylorgrams to differences in buffer composition between the protein and running buffer termed buffer mismatch, utilizing TDA with UV absorbance at 214 nm [27]. In this work, the present method had an obvious mismatch in the GuHCl concentration between the protein sample and denaturant solutions containing from 0 to 6 M GuHCl. However, it was seen that the mismatch did not affect the fluorescence consistently since it led to a fluorescence dip in the middle of the taylorgram for adalimumab and a fluorescence spike for HSA (Figure S2). This followed the trend observed in Figure 2 (main manuscript), where adalimumab fluorescence increased and HSA generally decreased at increasing GuHCl concentrations.

A) Adalimumab, 6 M GuHCl **B) HSA,** 3.5 M GuHCl

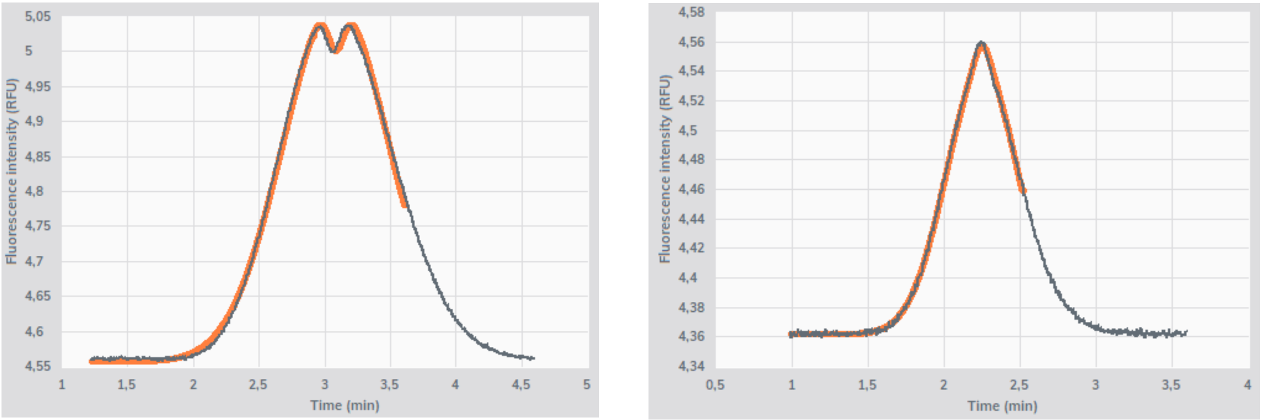

Figure S2: Taylorgrams demonstrating mismatch due to capillary mixing. A) Adalimumab (1 mg/mL, 6 M GuHCl, pH 4.0) where the mismatch generates a dip in fluorescence in the middle of the taylorgram B) HSA (1 mg/mL, 3.5 M GuHCl, pH 4.0) demonstrating the opposite behavior of adalimumab with a spike in fluorescence in the middle of the taylorgram. Orange lines represent two species fitting with one size fixed to 0.3 nm (R_h). Screenshots from Fida software.

It was hypothesized that insufficient mixing of GuHCl into the center of the protein injection was causing mismatch signals as this would lead to a locally lower GuHCl concentration and consequently locally less unfolding. Different injection volumes were therefore applied for HSA (Figure S3) which confirmed that larger injection volumes led to a higher degree of mismatch signals due to inadequate mixing of the protein plug into the adjacent denaturant zones. At the lowest injection volume (9 nL), the mismatch was practically eliminated (Figure S3). Applying a single species fit to the mismatch signals led to an overestimation of the R_h due to poor fitting of the signals which was least pronounced for the 9 nL injection (Table S1 and Figure S3). In this work, the mismatch phenomenon was mitigated by analyzing the taylorgrams with a two species fit, where one species was fixed to 0.3 nm as this fitted the data well, see Figure S2 and Table S1. This approach was considered appropriate because the hydrodynamic radii measured for HSA were comparable between capillary mixing and pre-incubation mode (Table S1).

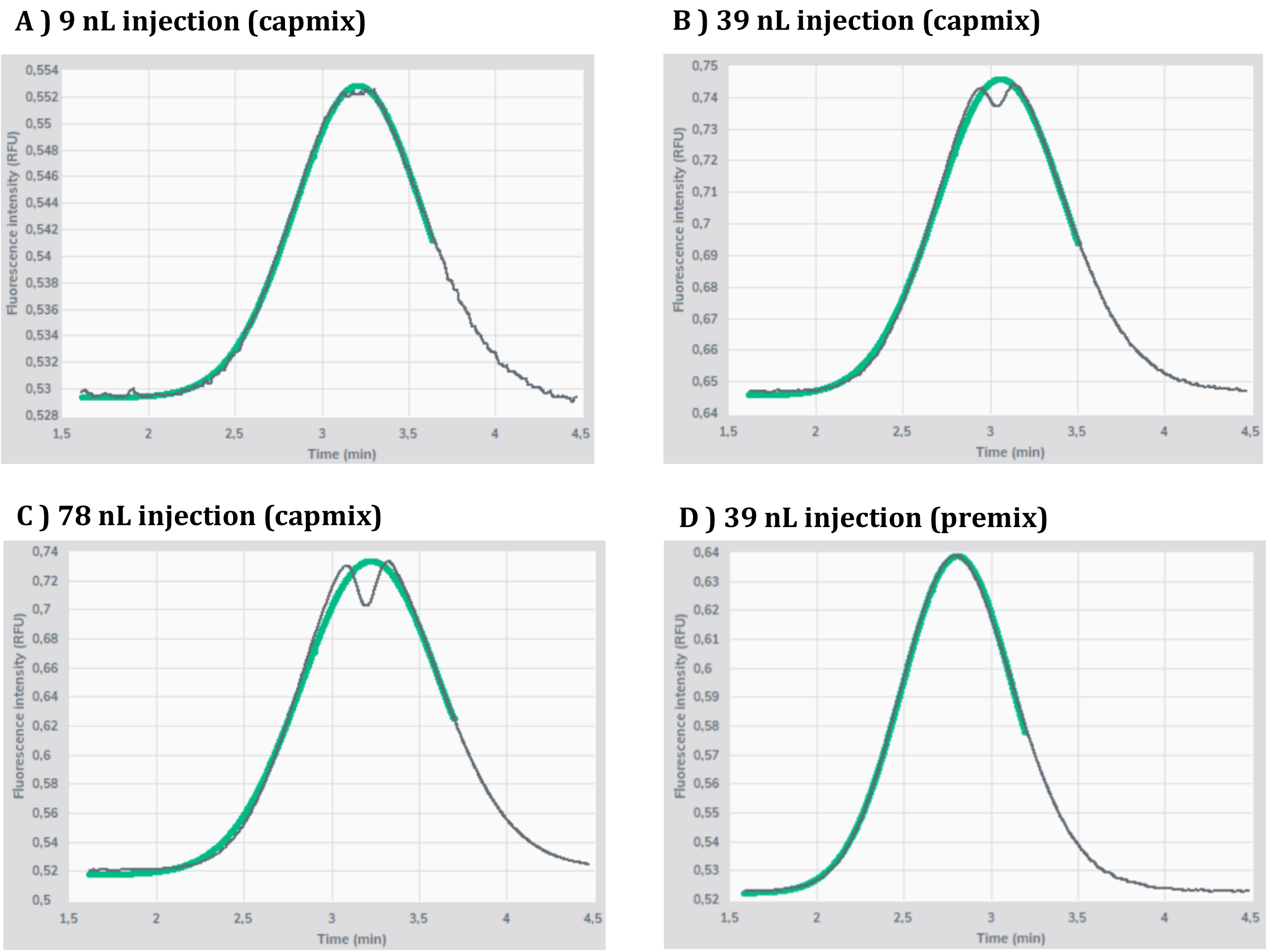

Figure S3: Taylorgrams of HSA (1 mg/mL) at pH 7.0 applying in-line capillary mixing with 6 M GuHCl at different injection volumes; A) 9 nL injection (10 s at 10 mbar), B) 39 nL injection (10 s at 50 mbar), and C) 78 nL injection (10 s at 100 mbar). D) HSA (1 mg/mL) pre-incubated with 5.5 nM GuHCl. Green lines represent one species fit (i.e., single Gaussian fit). Screenshots from Fida software.

Table S1: Hydrodynamic radii (± SD) of HSA (1 mg/mL) determined applying different mixing and injection modes as well as single and double species fitting procedures at 5.5 M GuHCl, pH 7.0.

	400 mbar, 39 nL (premix)	400 mbar, 9 nL (capmix)	400 mbar, 39 nL (capmix)	400 mbar, 78 nL (capmix)
Single species fit (nm)	6.13 ± 0.01	6.20 ± 0.04	6.69 ± 0.01	6.77 ± 0.08
Double species fit (nm)[a]	N/A	R_{h1}: 6.03 ± 0.04<br>R_{h2}: 0.30	R_{h1}: 6.13 ± 0.01<br>R_{h2}: 0.30	R_{h1}: 6.02 ± 0.07<br>R_{h2}: 0.30

[a]R_{h2} was fixed at 0.30 nm.

Unfolding kinetics of HSA

An advantage of the capillary mixing mode is that the reaction time with denaturant (GuHCl) inside the capillary can be precisely controlled by adjusting the flow rate accordingly. Thus in practice, the unfolding kinetics can be probed by modifying the mobilization pressure applied after injection of the protein sample (i.e., step 4 in the procedure section). Initially, a denaturation curve utilizing pre-incubated samples (> 1 h) was generated to set the scene for evaluating the unfolding kinetics of HSA (Figure S4). A small shift towards lower GuHCl concentrations was observed for the pre-incubated samples (premix) as compared to in-line capillary mixing (capmix), see Figure S4. This confirms that the full unfolding of HSA occurs on a longer timescale than the experimental timeframe of 2-4 min as expected [22].

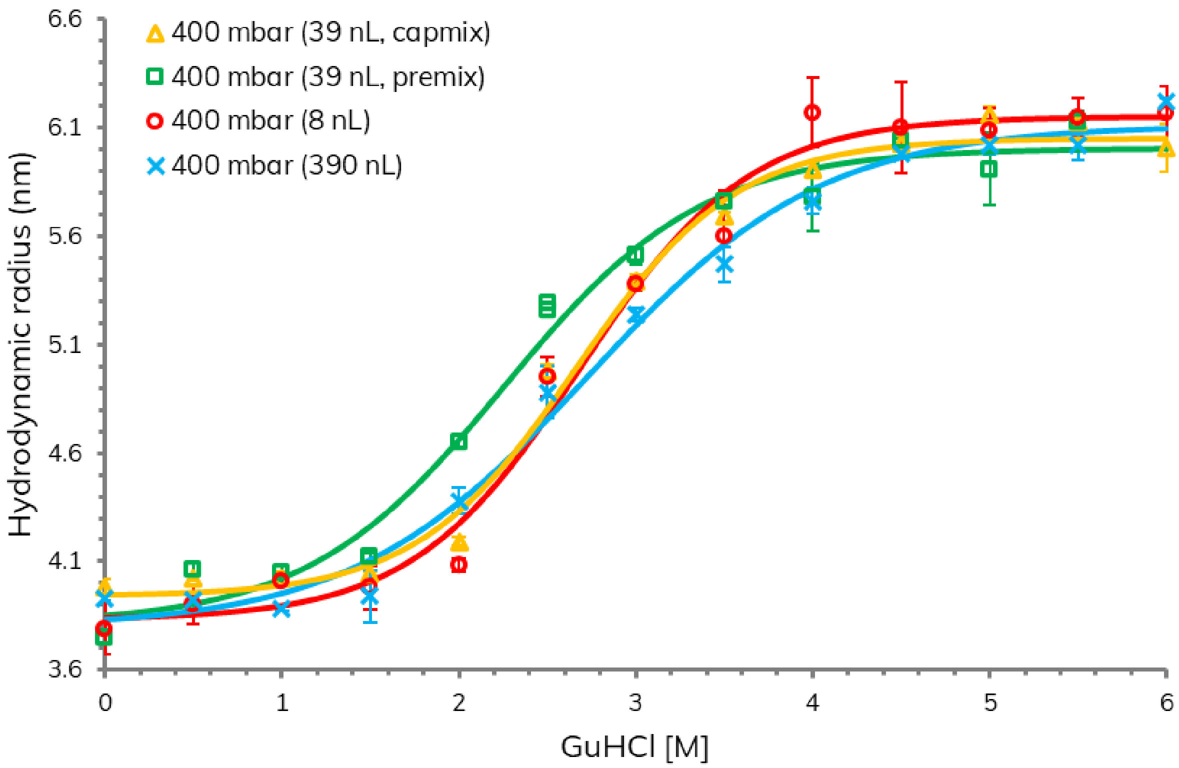

Figure S4: Hydrodynamic radius of HSA (1 mg/mL) as a function of GuHCl concentration (0 – 6 M) at pH 7.0 (25 °C) applying capillary mixing (yellow triangles), premixing (green squares) and injection volumes 8 nL (red circles, capmix), 39 nL (yellow triangles, green squares) and 390 nL (turquoise crosses, capmix). Solid lines represent fitting to the unfolding model (eq.1).

Varying the injection volume of HSA prior to in-line capillary mixing with GuHCl did also affect the denaturation curves (Figure S4) where a high injection volume of the protein sample (390 nL) led to a shift towards higher GuHCl concentrations and thus less effective unfolding of HSA. No differences were

seen between injection volumes 8 and 39 nL (Figure S4) suggesting there is an upper limit to efficient capillary mixing.

The incubation time with GuHCl was varied between 1 – 26 min corresponding to mobilization pressures between 50 – 800 mbar (Figure S5). Minor differences were observed between the different pressures such as offset and upper plateau for 50 and 100 mbar as compared to 200, 400, and 800 mbar (Figure S5). Interestingly, the viscosity was also slightly increased at 50 and 100 mbar (Table S2) at 6 M GuHCl. The viscosities were determined from the shift in residence time relative to the reference measurement in neat assay buffer (i.e., 0 M GuHCl) as previously described [16].

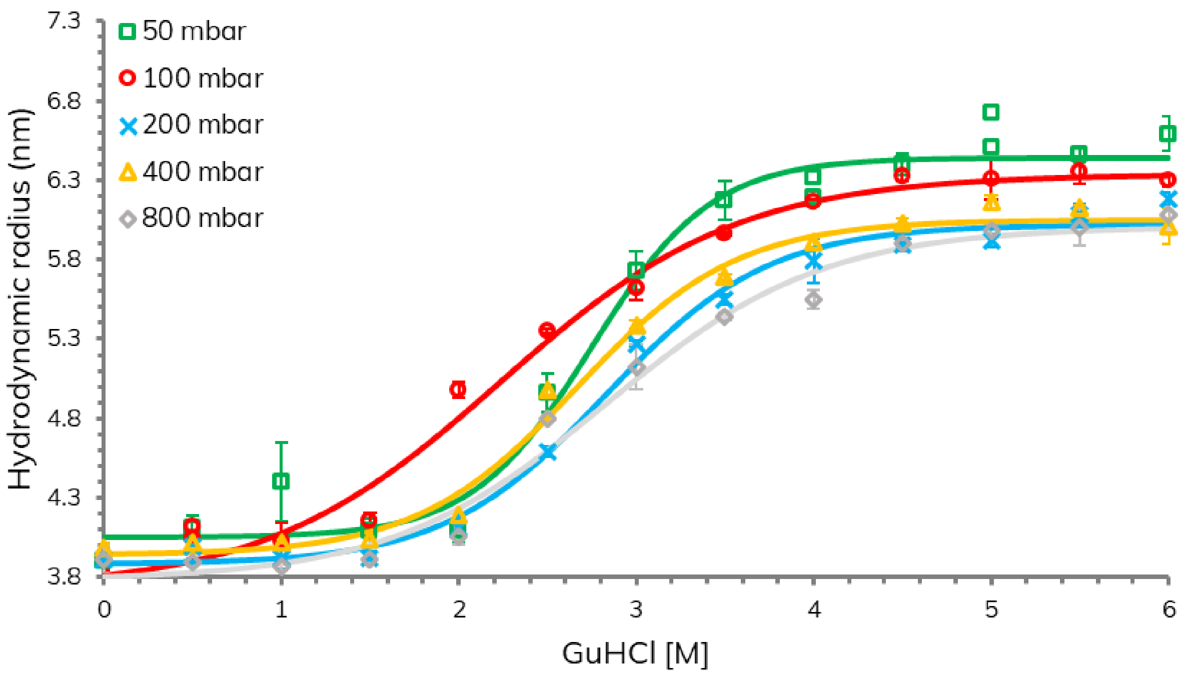

Figure S5: Hydrodynamic radius of HSA (1 mg/mL) as a function of GuHCl concentration (0 – 6 M) at pH 7.0 (25 °C) applying different mobilization pressures corresponding to incubation times of 1 – 26 min (800-50 mbar mobilization pressures). Solid lines represent fitting to the unfolding model (eq.1).

Table S2: Viscosity measurements at 50, 100, 200, 400, and 800 mbar (capmix) obtained from the shift in observed residence times at 6 M GuHCl, pH 7.0.

	50 mbar	100 mbar	200 mbar	400 mbar	800 mbar
Viscosity (mPa·s)	1.51 ± 0.01	1.48 ± 0.01	1.46 ± 0.01	1.45 ± 0.01	1.47 ± 0.00

The relative change in intrinsic fluorescence of HSA as a function of GuHCl concentration was comparable between in-line capillary mixing, premix, and high injection volume (390 nL), see Figure S6. However, the reliability on the fluorescence measurements was unsatisfactory when using low injection volumes (8 nL).

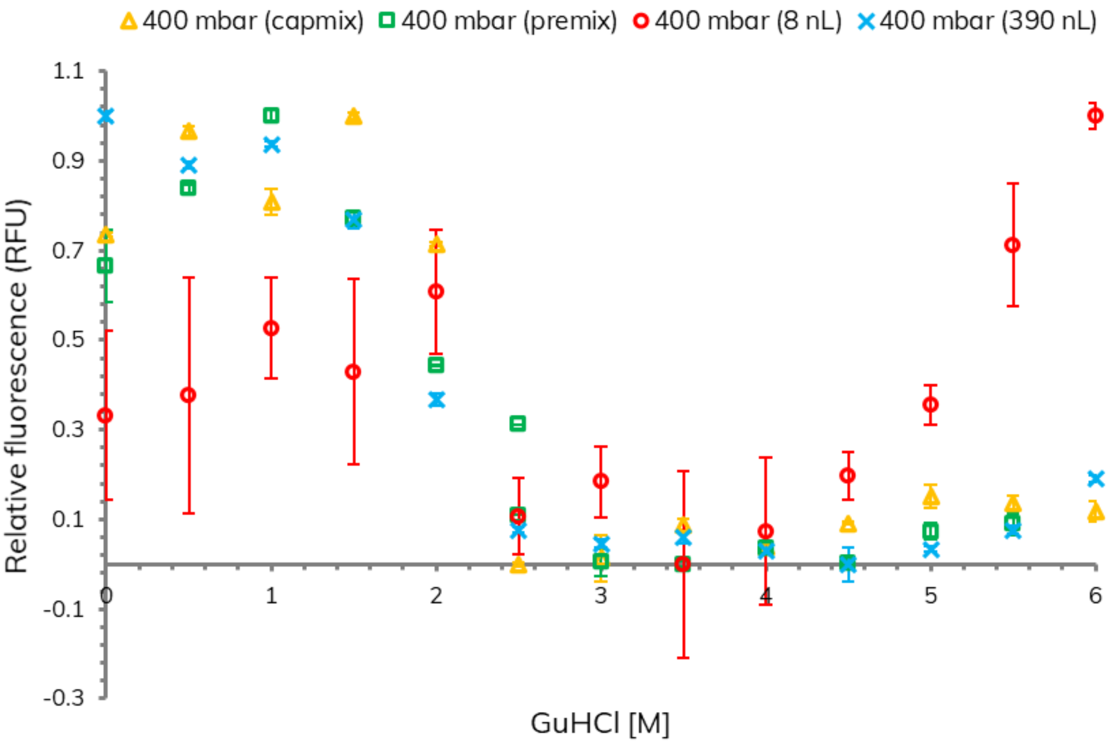

Figure S6: Normalized intrinsic fluorescence of HSA (1 mg/mL) as a function of GuHCl concentration (0 – 6 M) at pH 7.0 (25 °C) utilizing capillary mixing (yellow triangles), premixing (green squares) and injection volumes 8 nL (red circles, capmix), 39 nL (yellow triangles, green squares) and 390 nL (turquoise crosses, capmix).

Unfolding fitting model

Conformational stability assuming two states is given by:

$$F \rightleftharpoons U \qquad \text{(S1)}$$

where F and U are the folded and unfolded protein, respectively.

The unfolding equilibrium constant (K_U) can be expressed as:

$$K_U = \frac{[U]}{[F]} \qquad \text{(S2)}$$

where $[F]$ and $[U]$ are the concentration of folded and unfolded protein, respectively.

The unfolding reaction can be described by the standard free energy change ($\Delta G°$):

$$\Delta G° = -R \cdot T \cdot ln(K_U) \qquad \text{(S3)}$$

where R is the gas constant ($1.987 \cdot 10^{-3}$ kcal K^{-1} mol^{-1}) and T is the absolute temperature.

Assuming a linear dependency of $\Delta G°$ as a function of denaturant concentration ($[D]$):

$$\Delta G° = \Delta G°(H_2O) + m \cdot [D] \qquad \text{(S4)}$$

where $\Delta G°(H_2O)$ is the conformational stability in absence of denaturant, and m is the denaturant dependency (i.e., slope) on the standard free energy change.

Combining equation S3 and S4:

$$-R \cdot T \cdot ln(K_U) \;= \Delta G°(H_2O) + m \cdot [D] \qquad \text{(S5)}$$

S5 can be rearranged as:

$$K_U \;= e^{\left(\frac{\Delta G°(H_2O)+m\cdot[D]}{-R\cdot T}\right)} \qquad \text{(S6)}$$

The fraction of unfolded protein is termed x and given by:

$$x = \frac{[U]}{([U]+[F])} = \frac{[U]/[F]}{([U]/[F]+[F]/[F])} = \frac{K_U}{(K_U+1)} = \frac{e^{\left(\frac{\Delta G°(H_2O)+m\cdot[D]}{-R\cdot T}\right)}}{\left(e^{\left(\frac{\Delta G°(H_2O)+m\cdot[D]}{-R\cdot T}\right)}+1\right)} \qquad \text{(S7)}$$

The fraction of folded protein is thus 1 – x:

$$1 - x = \frac{(K_U+1)}{(K_U+1)} - \frac{K_U}{(K_U+1)} = \frac{1}{(K_U+1)} = \frac{1}{\left(e^{\left(\frac{\Delta G°(\mathrm{H_2O})+m\cdot[D]}{-R\cdot T}\right)}+1\right)} \quad \text{(S8)}$$

The apparent hydrodynamic radius of the protein (R_{app}) corresponds to the weighted average of folded and unfolded protein, which can be expressed as a function of the unfolded fraction (x):

$$R_{app} = ((R_{\mathrm{U}})^{-1} \cdot x + (R_{\mathrm{F}})^{-1} \cdot (1 - x))^{-1} \quad \text{(S9)}$$

where R_{app}, R_{U}, and R_{F} are the apparent (measured), unfolded, and folded hydrodynamic radii, respectively.

Combining equation S7-S9 provides the unfolding fitting model with four parameters:

$$R_{\mathrm{app}} = \left((R_{\mathrm{U}})^{-1} \cdot \left(\frac{e^{\left(\frac{\Delta G°(\mathrm{H_2O})+m\cdot[D]}{-R\cdot T}\right)}}{\left(e^{\left(\frac{\Delta G°(\mathrm{H_2O})+m\cdot[D]}{-R\cdot T}\right)}+1\right)} \right) + (R_{\mathrm{F}})^{-1} \cdot \left(\frac{1}{\left(e^{\left(\frac{\Delta G°(\mathrm{H_2O})+m\cdot[D]}{-R\cdot T}\right)}+1\right)} \right) \right)^{-1} \quad \text{(S10)}$$

When $\Delta G°(\mathrm{H_2O})$ and m are determined from fitting to S10, the denaturation midpoint (C_{m}) can be determined:

$$C_m = \frac{\Delta G°(\mathrm{H_2O})}{m} \quad \text{(S11)}$$
